# Contemporary Management of Popliteal Artery Aneurysms: A Comprehensive Review

**DOI:** 10.3390/medicina61112026

**Published:** 2025-11-13

**Authors:** Giulia Bertagna, Valentina Scarati, Nicola Troisi, Raffaella Berchiolli

**Affiliations:** Vascular Surgery Unit, Department of Translational Research and of New Technologies in Medicine and Surgery, University of Pisa, Cisanello Hospital, Via Roma 67, 56126 Pisa, Italy; giuliaberty.it@hotmail.it (G.B.); valentinascarati@gmail.com (V.S.); raffaella.berchiolli@unipi.it (R.B.)

**Keywords:** popliteal artery aneurysm, endovascular treatment, open surgical repair

## Abstract

*Background and Objectives*: current guidelines recommend surgical treatment for asymptomatic popliteal artery aneurysm > 20 mm in diameter, although without any suggestion about the preferred treatment choice. The two main treatment options are open surgical repair (OPAR) and endovascular repair (EPAR). Although ER has emerged as a promising technique due to being less invasive, OPAR remains the standard in many centers. The aim of the study is to report and compare outcomes of both endovascular and open repair of asymptomatic PAAs to provide an extensive overview of their current management. *Materials and Methods*: the present review was conducted in accordance with the Preferred Reporting Items for Review and Meta-Analyses (PRISMA) Guidelines. Preliminary searches were conducted on MEDLINE, Pubmed, Scopus, and Web of Science from January 2010 to September 2025. Articles were divided into three main groups based on the preferred treatment modality. Early outcomes were technical success, mortality, major adverse cardiovascular events (MACEs), and graft occlusion(s). In mid- and long-term periods, the evaluated outcomes were overall survival, amputation-free survival, primary patency, primary assisted patency, secondary patency, and freedom from reintervention. *Results*: 21 articles were identified for a total of 9760 patients and 10,062 limbs treated. Technical success was up to 100% for both OPAR and EPAR with low complication rates. Primary patency (79.8% vs. 63.8%; *p* = 0.012) and freedom from reintervention (82.2% vs. 68.4%; *p* = 0.021) seem to be better for OPAR than EPAR. Overall survival, amputation free-survival, and secondary patency rates are comparable between the two techniques. *Conclusions*: although endovascular repair has emerged as a safe and effective approach to treat elective PAAs, long-term data on a large scale are still lacking. Indeed, open surgical repair remains the milestone, due to excellent primary patency rates, regardless of the conduit used.

## 1. Introduction

Popliteal artery aneurysm (PAA) is the most common aneurysm among peripheral ones, and it is often associated with abdominal aortic aneurysms [1]. The real etiology of PAAs and general aneurysms is still unknown, although a combination of genetic defects and inflammatory processes seems to be responsible. In fact, atherosclerosis tends to increase flow turbulence distal to a stenosis, leading to a pathological dilation of the artery. Similarly, decreased wall strength associated with inflammatory cell infiltration results in aneurysm formation. Modifiable risk factors include smoking, hypercholesterolemia, hypertension, and diabetes, which lead to atherosclerosis. Non-modifiable risk factors include advanced age, male gender, white race, and family history of aneurysmal disease [2].

Among asymptomatic patients with PAAs, up to 24% will become symptomatic within 1–2 years and up to 68% will develop complications during the lifetime. Symptoms usually result from acute or chronic limb ischemia caused by distal embolism to the runoff tibial vessels. Patients presenting with acute limb ischemia (ALI) experienced an early amputation rate of 14%. On the other hand, patients presenting with chronic symptoms can be clinically indistinguishable from those with atherosclerotic arterial occlusive disease, ranging from intermittent claudication to chronic limb-threatening ischemia (CLTI). PAAs can also present with rupture, although this is rare, as well as compressive symptoms (venous congestion, leg swelling, deep vein thrombosis, and/or neuropathy) [3,4].

Current guidelines recommend surgical treatment for asymptomatic PAAs > 20 mm in diameter, although without specific recommendations about the treatment choice [5,6]. The two main options are open surgical repair (OPAR) via medial or posterior approach and endovascular repair (EPAR) with covered stents. Although endovascular PAA repair has emerged as a promising technique due to being less invasive, OPAR remains the gold standard in many centers [7,8]. On the other hand, EPAR has shown lower postoperative complications and shorter hospital stays compared to OPAR. This comes at the cost of inferior primary patency up to 3 years [9].

There are few studies in the current literature evaluating the durability in the long term of endovascular treatment for asymptomatic PAAs. A few years ago, Shah et al. [10] described no significant differences in primary and secondary patency between OPAR and EPAR during long-term follow-up. However, this analysis was based on a small sample size, and the cohort was heterogenous, so that the OPAR group had a significantly higher number of patients presented with ALI. Recently, the PARADE multicenter study, based on highly selected patients, clarified the complementary role of open and endovascular repair for asymptomatic PAAs [11,12].

Therefore, the aim of the study is to report and compare outcomes of both endovascular and open surgical treatment of asymptomatic PAAs to provide an extensive overview of current management in the framework of a review.

## 2. Materials and Methods

The protocol and methodology of this review was conducted and reported in accordance with the Preferred Reporting Items for Review and Meta-Analyses (PRISMA) statement and the recommendations by Koelemay and Vermeulen [13,14].

Before performing the entire search strategy, the Patient, Intervention, Comparison, Outcome (PICO) framework [15] was established to specify the objective of the study: patient (“popliteal artery aneurysm”, “asymptomatic”); intervention (“endovascular repair”, “endovascular treatment”, “EPAR”, “open surgical repair”, “open repair”, “OPAR”); comparison (open surgical repair vs. endovascular treatment); and outcome (“primary patency”, “primary assisted patency”, “secondary patency”, “limb loss rate”, “reintervention rate”).

The literature search strategy was independently performed by two authors (V.S, G.B.). In the event of disagreement, a third author (N.T.) was consulted to make the final judgement and provide consensus. Preliminary searches were conducted on Medical Literature Analysis and Retrieval System Online (MEDLINE), Pubmed, Scopus, and Web of Science, from 1st January 2010 and 1st September 2025, and an English language filter was applied. The Society of Vascular Surgery (SVS) guidelines were also reviewed.

A combination of controlled vocabulary and free text terms was used to investigate the databases.

In particular, the following search strategies were used on each one of the databases:

*“Popliteal artery aneurysm AND endovascular treatment” (textword).*

*“Popliteal artery aneurysm AND open surgery” (textword).*

*“Popliteal artery aneurysm AND (endovascular treatment OR endovascular repair)” (textword).*

*“Popliteal artery aneurysm AND (open surgery OR open surgical repair)” (textword).*

Narrative reviews and articles with few or incomplete data were excluded. Studies updated or duplicated were excluded too. Inclusion criteria involved the management of asymptomatic PAAs treated by open or endovascular repair. When possible, abstracts were reviewed online through specific websites and suitable articles downloaded entirely for data extraction. If abstracts were not available, a full copy of the article was assessed.

Exclusion criteria were as follows:

- Reason 1: narrative review without significant data
- Reason 2: studies with few or incomplete data
- Reason 3: studies updated or duplicated
- Reason 4: symptomatic PAAs (for ALI)
- Reason 5: ruptured PAAs
- Reason 6: clinical cases or series

Then, articles were divided into three main groups based on the preferred treatment modality to improve the readiness of the reported outcomes, as follows:

- Group 1: studies reporting early and/or long-term outcomes after OSR only;
- Group 2: studies reporting early and/or long-term outcomes after EPAR only;
- Group 3: studies reporting and comparing early and/or long-term outcomes after both OPAR and EPAR.

**Figure 1:**
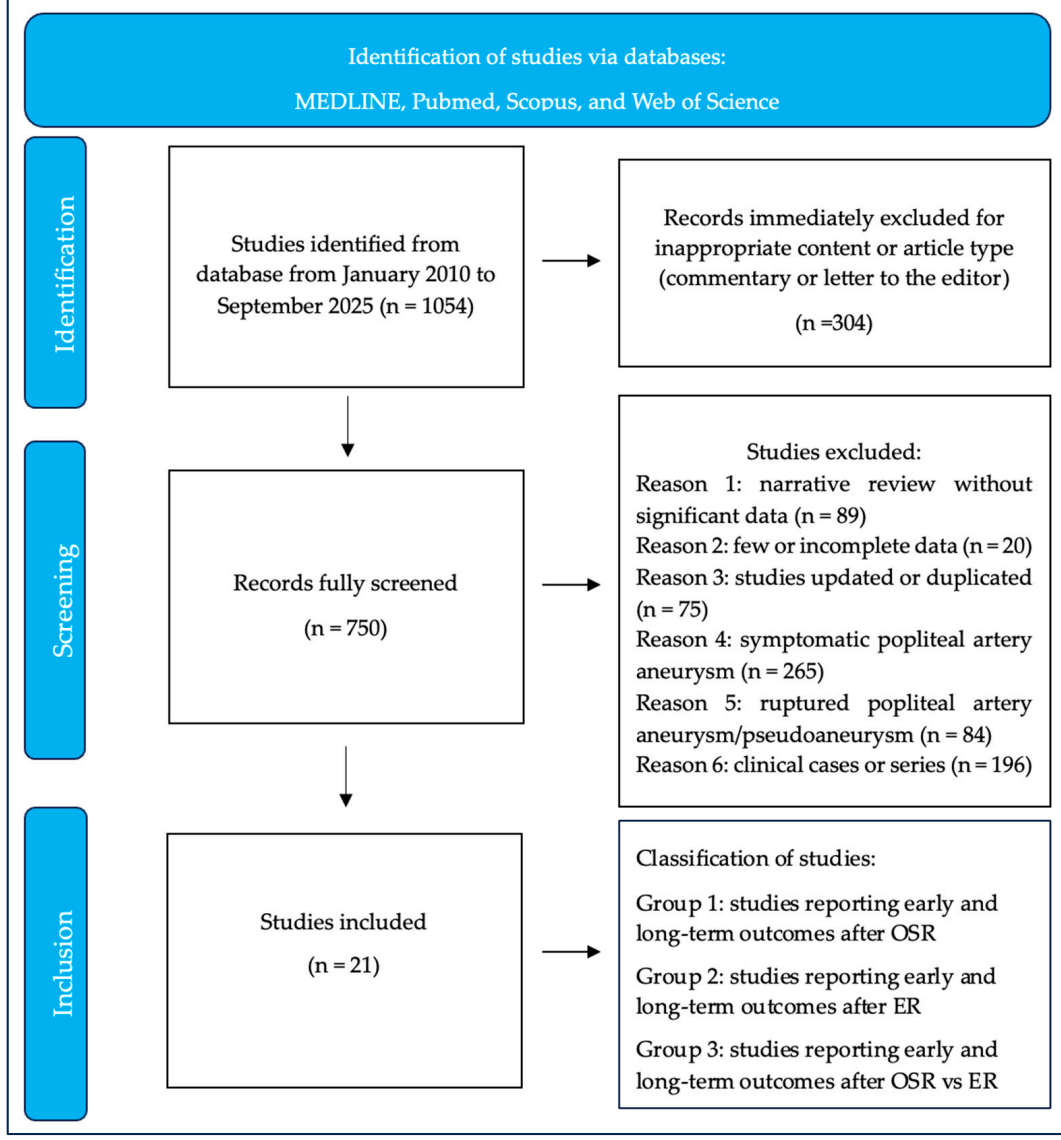
Shows the research flowchart based on the PICOS approach.

The flow-chart of the research is reported in Figure 1 based on the PICOS approach.

### 2.1. Definitions

Technical success was defined as complete exclusion of the aneurysm with surgical or endovascular graft patency with direct flow into at least one below-the-knee (BTK) vessel.

Primary patency was defined as no evidence of restenosis of the graft during follow-up, namely, a peak systolic velocity ratio (PSVR) ≥ 2.5 or graft occlusion, based on duplex ultrasound (DUS).

Primary assisted patency was defined as time to reintervention to maintain patency. Secondary patency was defined as graft patency maintained by repeat intervention after complete graft occlusion.

Freedom from reintervention was defined as the absence of reintervention in the index limb during the follow-up strictly related to the index procedure.

Amputation free survival was defined as freedom from any amputation ipsilateral to the target lesion above the level of the ankle joint [11].

### 2.2. Outcomes Measures

Early outcomes included technical success, mortality, major adverse cardiovascular events (MACEs), and graft occlusion(s).

In mid- and long-term periods, the evaluated outcomes were overall survival, amputation-free survival, primary patency, primary assisted patency, secondary patency, and freedom from reintervention.

### 2.3. Statistical Analysis

Continuous data were expressed as the mean values. Categoric data were expressed as percentages. The Pearson chi-square test was used to compare values between groups, based on the nature of data and variables.

Statistical significance was defined at the *p* < 0.05 level.

Statistical analysis was performed using SPSS software (version 24.0 for Apple; IBM Corporation, Armonk, NY, USA).

## 3. Results

A total of 1054 articles were identified after primary database searching (Figure 1). After inappropriate report removal, 750 articles were screened. Altogether, 729 studies were not eligible based on title and abstract screening and availability. The two most important reasons for exclusion were the lack of a separate investigation for asymptomatic patients specifically and the lack of relevant data because of a small sample source. Finally, 21 articles were included in the present analysis, for a total of 9760 patients and 10,062 limbs treated, respectively.

Patients included had asymptomatic PAAs and were treated with open repair via medial or posterior approach, using different types of conduits (autologous vein or prosthetic), with endovascular repair with covered stents (mainly Viabahn peripheral endograft—W. L. Gore and Associates, Inc., Flagstaff, AZ, USA), or with both open and endovascular treatment. In this latter case, outcomes were compared between the two groups.

### 3.1. Open Popliteal Aneurysm Repair (OPAR)

The majority of patients in this group were males (97%, range 95–99) with a mean age of 68.5 ± 9. Patients presented with all risk factors for atherosclerotic disease: history of smoking (61.3%), arterial hypertension (57.3%), hyperlipidemia (52%), and ischemic heart disease (26.7%). Open surgical repair seems to offer excellent results, with 100% technical success for all the studies selected. Mortality in the perioperative period is low, and only one paper reported a 30-day mortality of 1%, which was not procedure related. The same study shows relatively high rates of graft occlusion up to 7.7% but successfully treated with early reinterventions. More recent studies focusing on the type of graft used (autologous vein vs. prosthetic) did not show any statistically significant differences in early results.

Concerning long-term outcomes, OPAR demonstrates good durability over an extended period [16,17,18,19]. Indeed, Dorigo et al. [16] reported 13-year outcomes in terms of amputation-free survival, primary patency, secondary patency, and freedom from reintervention of 45%, 55.1%, 68%, and 62%, respectively. Recently, the study by Troisi et al. [18], comparing patients treated with great saphenous vein (GSV) vs. ePTFE, demonstrated better primary patency (GSV 89.5% vs. 76.2% ePTFE; *p* = 0.007) and freedom from reintervention (GSV 92.8% vs. 80.6% ePTFE; *p* = 0.011) rates for patients treated with autologous veins. Other authors did not find any differences between types of conduits in terms of amputation-free survival, without reporting any additional data [19].

Table 1 shows in detail early and long-term outcomes of all studies selected concerning OPAR.

### 3.2. Endovascular Popliteal Aneurysm Repair (EPAR)

Concerning demographic features, patients were predominantly males (95.3%, range 98.1–93) with a mean age of 77.1 ± 8. Smoking habit was as frequent as up to 80% (range 40–82) of patients enrolled. Furthermore, 77.6% of patients suffered from hypertension, 58.8% from hypercholesterolemia, and 37.3% had coronary artery disease.

Patients undergoing EPAR experienced high rates of technical success up to 100%, similar to surgically treated patients. Mortality in perioperative period is negligible with a 30-day mortality of 0.6%, with rates of MACEs of 1.2%. In terms of early graft occlusion, data are heterogenous, ranging from 0% to 16%.

Long-term results are satisfactory, although with lower overall patency rates compared to the open approach. The largest study on 326 patients reported rates of primary patency and freedom from reintervention at 5 years of 65.8% and 70.5%, respectively. van Leeuwen, GL. et al. [20] showed lower rates for both the outcomes considered (54% and 69%, respectively). On the other hand, amputation-free survival rates are acceptable in all studies selected, varying from 96.5% to 100%.

Table 2 illustrates outcomes after EPAR for all studies included.

### 3.3. Open vs. Endovascular Popliteal Aneurysm Repair (OPAR vs. EPAR)

Eleven articles met the inclusion criteria comparing OPAR and EPAR in asymptomatic patients. Most patients were males (97%, range 96–99) with a higher age in the EPAR group compared to OPAR (75.1 vs. 69.9, *p* < 0.001). Remaining preoperative demographic features were comparable between the two groups, with all risk factors of atherosclerotic disease.

All studies reported high rates of technical success for both treatments. Graft occlusions are more frequent in EPAR group in the immediate postoperative period, up to 14.2% of the cases. Patients in open group experienced higher rates of MACEs (OPAR 2.3% vs. 0.9% EPAR; *p* = 0.04).

During long-term follow-up period, rates of overall survival are similar between open and endovascular in almost all reports, apart from two studies that showed a slight difference in favor of OPAR (*p* = 0.050; *p* = 0.04; *p* = 0.045). These data were reported to be associated with the younger age of surgically treated patients. Troisi et al. [11] demonstrated better primary patency rates in open group (OPAR 79.8% vs. 63.8% EPAR; *p* = 0.012), similarly to what was reported by Cervin et al. [27] (OPAR 89% vs. 67.4% EPAR; *p* = < 0.001). The POPART registry showed even worse primary patency for EPAR (74.2% vs. 29.1%; *p* = < 0.005). Several authors demonstrated higher secondary patency and freedom from reintervention rates with open surgery [8,27,28,29,30].

Table 3 illustrates all studies reporting and comparing early and long-term outcomes after OPAR and EPAR.

## 4. Discussion

The present study shows comparable outcomes between open and endovascular repair for asymptomatic PAAs in the early and mid-term period, with low complication rates. Despite these promising data, open surgery remains the cornerstone, thanks to excellent patency rates over an extended period. Even though early graft occlusions have been reported up to 7.7%, these cases were successfully managed by early reintervention. Indeed, OPAR allows the achievement of satisfactory outcomes in terms of primary patency and amputation-free survival up to 13 years of follow up [16].

Current guidelines do not recommend any specific treatment modalities nor type of conduit, so that results of open surgery with different grafts are heterogenous. Some authors did not find any difference between venous and prosthetic conduits, while Troisi et al. [18] demonstrated better primary patency and freedom from reintervention in patients treated with a single segment great saphenous vein compared to those treated with ePTFE. Furthermore, the absence of preoperative antiplatelet therapy and dialytic treatment seemed to be negative predictors of primary patency in ePTFE group. At present, very few studies discuss the importance of medical management optimization but are mainly focused on surgical strategies. On the other hand, it is well known the role of antiplatelet agents in reducing the risk of thrombogenic events before and after treatment, as well as some anticoagulation schemes [36]. The PARADE study shows that patients who underwent OPAR received more direct oral anticoagulant (DOAC) postoperatively than patients who underwent EPAR, who instead were given dual antiplatelet therapy (DAPT). This can be explained by a trend toward DOAC in patients who received a vein conduit but also by the need for this medical therapy due to other comorbidities. On the other hand, DAPT was more suitable for patients who were implanted with a stentgraft. Indeed, recent studies show that up to 58.5% of patients who underwent EPAR were treated with DAPT and up to 15% for at least a six-week period [20]. These data underline the importance of pre- and postoperative medical optimization, regardless of the graft used. Therefore, the choice of different types of conduits should be targeted to patient-specific features.

The endovascular approach represents a minimally invasive technique with low 30-day mortality and low complication rates in terms of MACEs. In the perioperative period, very few patients experienced a major cardiovascular event, represented by acute myocardial infarction and fatal dysrhythmia in almost all cases (cardiovascular deaths).

Technical success rates are high and comparable to those of open repair, while graft occlusions are more frequent with endovascular repair. In support of these data, it should be noted that endovascular repair led to lower rates of primary patency, so that more reinterventions are necessary to restore flow, and freedom from reintervention is lower accordingly. Despite inferior primary patency rates in endovascularly treated patients, the amputation-free survival is satisfactory and comparable with open surgery. These data support the choice of using endovascular repair in highly selected cases. In particular, EPAR seems to offer a valid option in patients with multiple comorbidities and unfit for open surgery, where the saying “less is more” holds true. Obviously, this category of patient needs to have suitable anatomy and sufficient runoff status in order to maintain good patency rates and prevent stent occlusion.

Indeed, even looking at the studies comparing OPAR and EPAR, the minimally invasive approach offers greater results in terms of perioperative morbidity and mortality. As aforementioned, mortality and MACEs are negligible after EPAR. Major adverse cardiovascular events are similar between OPAR and EPAR. The open approach leads to a higher proportion of chronic heart failure, acute myocardial infarction, fatal dysrhythmia, and strokes. This is intuitive considering the different impact of endovascular and open surgery on patients’ clinical status. However, recent evidence demonstrates better survival rates in surgically treated patients. Although the two groups (OPAR and EPAR) were homogenized in terms of preoperative comorbidities and medical treatment, the mean age resulted as statistically significant in almost all studies included. This can be explained by the older age of patients who underwent EPAR. Indeed, in these patients, a careful evaluation of the risk–benefit balance, in terms of life-expectancy and comorbidities combined with anatomical assessment, plays a pivotal role. On the other hand, it has to be mentioned that all data included come from open-label analysis, so that a certain selection bias cannot be addressed. This may justify the choice of the endovascular approach first in elderly and unfit patients with suitable anatomy, in terms of safe landing zones and aneurysm location.

Again, open surgery allows the achievement of superior primary patency with lower rates of reintervention compared to the endovascular approach. In addition, significantly better secondary patency rates have been reported, outlining that restoring an in-line flow after open repair could be more effective [27,29]. The recent PARADE study, based on a larger cohort, further states the longer durability of OPAR. This analysis highlights that outcomes following surgical treatment for asymptomatic PAAs < 60 mm in length via the posterior approach are almost comparable with those achieved via endovascular repair using covered stents up to 5-years of follow up. Different to what was previously reported, this analysis was based on highly selected patients so that results could be reproducible in similar settings. However, open surgery with the posterior approach seems to be associated with nerve injuries compared to the other available techniques. These data reflect the real-word nature of this multicentric experience, although surgical expertise and good patient selection may overcome these complications.

Eventually, all the studies examined reveal comparable short-term outcomes between endovascular and open repair, regardless of the type of approach (medial or posterior) and type of conduit (venous or prosthetic), although the great saphenous vein seems to be the conduit of choice in patients with good vein heritage over an extended period. During long-term follow up, open repair offers better patency rates combined with low rates of both reintervention and limb loss. To summarize, our review suggests that younger age, the availability of single-segment GSV, challenged runoff vessels (two or only one), and lower operative risk are features in favor of the OPAR approach. However, greater equipoise for EPAR is present with elderly age, an insufficient GSV, the presence of satisfactory landing zones and tibial runoff with all three or at least two vessels, and higher operative risk.

This study has several limitations that should be mentioned. First, there are few studies comparing OPAR and EPAR in highly selected patients, so that results appear heterogenous. Second, in this review, patients treated with different types of approach (medial or posterior) and types of conduits (autologous vein or prosthetic graft) were pooled together, reducing the generalizability of results. Then, the majority of data have been extracted from retrospective and relatively small cohorts, reducing the power of the statistical analysis.

## 5. Conclusions

Endovascular repair for asymptomatic PAAs has emerged as a safe, less invasive, and effective approach with similar early and mid-term outcomes compared to open surgical repair. Current guidelines do not recommend any specific treatment option. However, this paper demonstrates poorer primary patency rates for endovascular repair compared to open surgery, with higher rates of reintervention in the long-term period. Indeed, open repair remains the milestone of treatment. For this reason, patients should be offered the best treatment between the two options, considering their anatomy and fitness. Eventually, further studies on a larger sample size and randomized may define the superiority of one treatment over another or their complementary role.

## Figures and Tables

**Table 1:** Studies reporting outcomes after open surgical repair of PAAs.

Study	N° of Patients (Limbs Treated)	Early and Mid-Term Outcomes	%			Long-Term Outcomes	%		
Dorigo, W. et al. [16] J. Vasc. Surg. 2015	196 (234)	-technical success -mortality -MACEs -graft occlusion(s)	100% 1% - 7.7%			Estimated 13-year: -overall survival -amputation-free survival -primary patency -primary assisted patency -secondary patency -freedom from reintervention	50.8% 45% 55.1% - 68% 62%		
Chang, H. et al. [17] Ann. Vasc. Surg. 2021	1065 (1146)	-technical success -mortality -MACEs -graft occlusion(s)	100% - - -			-overall survival -amputation-free survival -primary patency -primary assisted patency -secondary patency -freedom from reintervention	GSV - - 87% - 96% -	PC - - 91% - 95% -	*p* - - NS - NS -
Troisi, N. et al. [18] J. Vasc. Surg. 2025	525 (525)	-technical success -mortality -MACEs -graft occlusion(s)	GSV 100% 0.4% 1.2% 2.8%	ePTFE 100% 0% 1.1% 0.8%	*p* - 0.480 0.601 0.081	-overall survival -amputation-free survival -primary patency -primary assisted patency -secondary patency -freedom from reintervention	GSV 84.7% 99.1% 89.5% - 94.9% 92.8%	ePTFE 86.1% 99.6% 76.2% - 89.4% 80.6%	*p* 0.097 0.567 0.007 - 0.068 0.011
Kim, Y. et al. [19] Ann. Vasc. Surg. 2024	101 (101)	-technical success -mortality -MACEs -graft occlusion(s)	100% 0 - -			-overall survival -amputation-free survival -primary patency -primary assisted patency -secondary patency -freedom from reintervention	GSV 98% 80.8% - - - -	PC 98% 76.7% - - - -	*p* - 0.47 - - - -

GSV: great saphenous vein; PC: prosthetic conduits; ePTFE: polytetrafluoroethylene; MACEs: major adverse cardiovascular events.

**Table 2:** Studies reporting outcomes after EPAR.

Study	N° of Patients (Limbs Treated)	Early and Mid-Term Outcomes	%	Long-Term Outcomes	%
Guzzardi, G. et al. [21] Radiol. Med. 2019	48 (48)	-technical success -mortality -MACEs -graft occlusion(s)	100% 0 - 10.4%	-overall survival -amputation-free survival -primary patency -primary assisted patency -secondary patency -freedom from reintervention	100% 100% 70.8% - 89.6% 75%
Speziale, F. et al. [22] Ann. Vasc. Surg. 2015	53 (53)	-technical success -mortality -MACEs -graft occlusion(s)	100% 0 3.8% 0	-overall survival -amputation-free survival -primary patency -primary assisted patency -secondary patency -freedom from reintervention	96.2% 100% 73.2% - 92.4% 81.1%
van Leeuwen, GL. et al. [20] Eur. J. Vasc. Endovasc. Surg. 2025	105 (123)	-technical success -mortality -MACEs -graft occlusion(s)	98.4% 0 - 3.3%	-overall survival -amputation-free survival -primary patency -primary assisted patency -secondary patency -freedom from reintervention	77% 98.4% 54% 58% 73% 69%
Troisi, N. et al. [23] J. Vasc. Surg. 2025	326 (326)	-technical success -mortality -MACEs -graft occlusion(s)	99.4% 0.6% 1.2% 3.7%	-overall survival -amputation-free survival -primary patency -primary assisted patency -secondary patency -freedom from reintervention	82.7% 98.2% 65.8% 86.5% 84.9% 70.5%
Golchehr, B. et al. [24] Eur. J. Vasc. Endovasc. Surg. 2016	70 (72)	-technical success -mortality -MACEs -graft occlusion(s)	100% 0 0 0	-overall survival -amputation-free survival -primary patency -primary assisted patency -secondary patency -freedom from reintervention	90% 100% 69% 74% 76% 82%
Midy, D. et al. [25] J. Vasc. Surg. 2010	50 (57)	-technical success -mortality -MACEs -graft occlusion(s)	98.2% 0 - 16%	-overall survival -amputation-free survival -primary patency -primary assisted patency -secondary patency -freedom from reintervention	91% 96.5% 87.5% - 83% 96.2%
Piazza, M. et al. [26] Eur. J. Vasc. Endovasc. Surg. 2014	42 (46)	-technical success -mortality -MACEs -graft occlusion(s)	98% 0 - 5%	-overall survival -amputation-free survival -primary patency -primary assisted patency -secondary patency -freedom from reintervention	84% 98% 76% - 82% 63%

**Table 3:** Studies reporting and comparing outcomes of OPAR vs. EPAR.

Study	N° of Patients (Treated Limbs)	Early and Mid-Term Outcomes	OPAR	EPAR	*p*	Long-Term Outcomes	OPAR	EPAR	*p*
Troisi, N. et al. [11] Eur. J. Vasc. Endovasc. Surg. 2025	605 (605)	-technical success -mortality -MACEs -graft occlusion(s)	100% 0.7% 1.6% 2.3%	98.8% 0.6% 0.6% 4.2%	- 0.18 0.31 0.15	-overall survival -amputation-free survival -primary patency -primary assisted patency -secondary patency -freedom from reintervention	84.4% 99% 79.8% - 90.7% 82.2%	79.4% 98.% 63.8% - 85.2% 68.4%	0.050 0.73 0.012 - 0.25 0.021
Galiñanes, EL. et al. [28] Vasc. Endovascular. Surg. 2013	2962 (2962)	-technical success -mortality -MACEs -graft occlusion(s)	- - 2.3% -	- - 0.9% -	- - 0.04 -	-overall survival -amputation-free survival -primary patency -primary assisted patency -secondary patency -freedom from reintervention	- - - - - 95.4%	- - - - - 88.2%	- - - - - 0.001
Del Tatto, B. et al. [29] Ann. Vasc. Surg. 2018	126 (153)	-technical success -mortality -MACEs -graft occlusion(s)	- - - 4.9%	- - - 12%	- - - NS	-overall survival -amputation-free survival -primary patency -primary assisted patency -secondary patency -freedom from reintervention	97.7% 89.5% 77.8% 85% 92.8% -	92.4% 97.7% 44.4% 69.3% 84.3% -	- - **0**.02 - 0.048 -
Ripepi, M. et al. [30] Ann. Vasc. Surg. 2025	120 (143)	-technical success -mortality -MACEs -graft occlusion(s)	- 0 - 1.4%	- 0 - 5.5%	- NS - 0.2	-overall survival -amputation-free survival -primary patency -primary assisted patency -secondary patency -freedom from reintervention	94.3% 100% 81% - 90.6% 95.7%	91.8% 99% 64.1% - 88.4% 70.9%	0.04 0.5 0.01 - 0.3 0.02
Cervin, A. et al. [27] Eur. J. Vasc. Endovasc. Surg. 2015	499 (592)	-technical success -mortality -MACEs -graft occlusion(s)	100% 0 - -	100% 0 - -	NA NA - -	-overall survival -amputation-free survival -primary patency -primary assisted patency -secondary patency -freedom from reintervention	98.8% 97.8% 89% - 93.5% -	94.6% 92.3% 67.4% - 83.7% -	0.045 0.048 <0.001 - 0.026 -
Eslami, MH. et al. [8] J. Vasc. Surg. 2015	390 (390)	-technical success -mortality -MACEs -graft occlusion(s)	100% 0 0.6% -	100% 0 1.8% -	NA NA NS -	-overall survival -amputation-free survival -primary patency -primary assisted patency -secondary patency -freedom from reintervention	95.5% 91.8% 95.9% - 91.1% -	98.2% 77.2% 92.3% - 76.6% -	0.32 0.008 0.368 - 0.006 -
Dorigo, W. et al. [31] Scand J. Surg. 2018	309 (309)	-technical success -mortality -MACEs -graft occlusion(s)	100% 0 7% 1.7%	100% 1.7% - 3.5%	NA 0.3 - 0.4	-overall survival -amputation-free survival -primary patency -primary assisted patency -secondary patency -freedom from reintervention	89.5% 86.4% 64% - 71% 76%	94% 94% 72% - 74% 65.5%	0.4 0.3 0.8 - 0.9 0.2
Leake, AE. et al. [32] J. Vasc. Surg. 2016	156 (186)	-technical success -mortality -MACEs -graft occlusion(s)	100% 1.6% - 1.8%	100% 0 - 0	NA 0.67 - NA	-overall survival -amputation-free survival -primary patency -primary assisted patency -secondary patency -freedom from reintervention	96% 96.8% 79.5% 83.7% 85% 96.8%	96% 100% 73.2% 76.3% 83% 91.1%	NS 0.23 0.630 0.495 0.963 0.28
Pulli, R. et al. [33] Ann. Vasc. Surg. 2012	59 (64)	-technical success -mortality -MACEs -graft occlusion(s)	- 4.5% - 4.5%	100% 4.7% - 14.2%	- 0.9 - 0.2	-overall survival -amputation-free survival -primary patency -primary assisted patency -secondary patency -freedom from reintervention	96.5% 92.7% 78.1% - 81.6% 79%	96.5% 95% 59.4% - 78.4% 61.5%	- 0.7 0.1 - 0.9 0.2
Satam, K. et al. [34] J. Vasc. Surg. 2025	1159 (1159)	-technical success -mortality -MACEs -graft occlusion(s)	100% 1.8% - -	100% 1.7% - -	NA NA - -	-overall survival -amputation-free survival -primary patency -primary assisted patency -secondary patency -freedom from reintervention	97% 97.1% - - - 96%	97% 98% - - - 91.1%	NA 0.76 - - - 0.56
Jung, G. et al. [35] J. Vasc. Surg. 2022	794 (768)	-technical success -mortality -MACEs -graft occlusion(s)	- 0.5% 0.8% 0.4%	- 1.9% 0.9% 1.9%	- 0.143 0.591 0.655	-overall survival -amputation-free survival -primary patency -primary assisted patency -secondary patency -freedom from reintervention	92% - 74.2% - - -	92% - 29.1% - - -	NA - <0.005 - - -

## Data Availability

No new data were created or analyzed in this study.
